# Endoscopic submucosal tunneling resection via a transantral approach for duodenal bulb gastrointestinal stromal tumor

**DOI:** 10.1055/a-2844-3055

**Published:** 2026-05-08

**Authors:** Junye Liufu, Yuchen Yang, Jinwen Huang, Chenguang Xu, Pi Liu

**Affiliations:** 1 Department of Gastroenterology 380381 The Peopleʼs Hospital of Longhua ShenzhenShenzhenChina

**Fig. 1:**
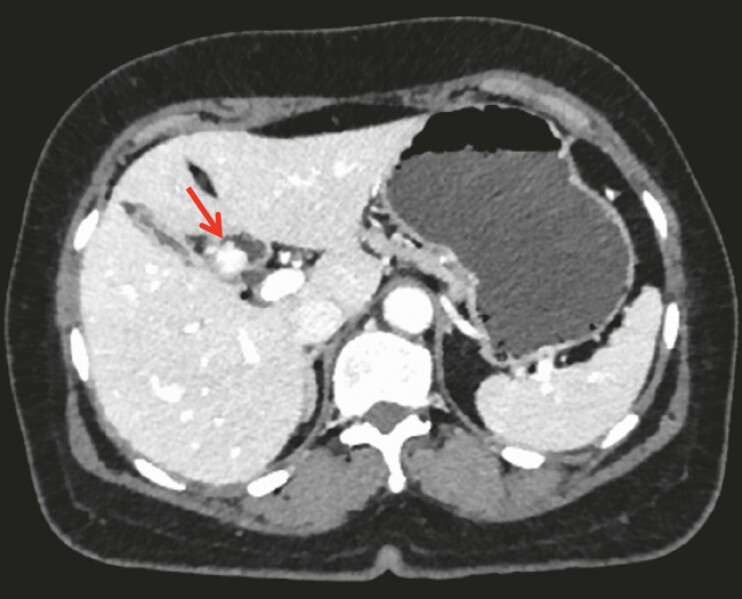
A contrast-enhanced CT image revealing a well-defined, enhancing nodule at the superior aspect of the duodenal bulb (arrow). CT, computed tomography.

**Fig. 2:**
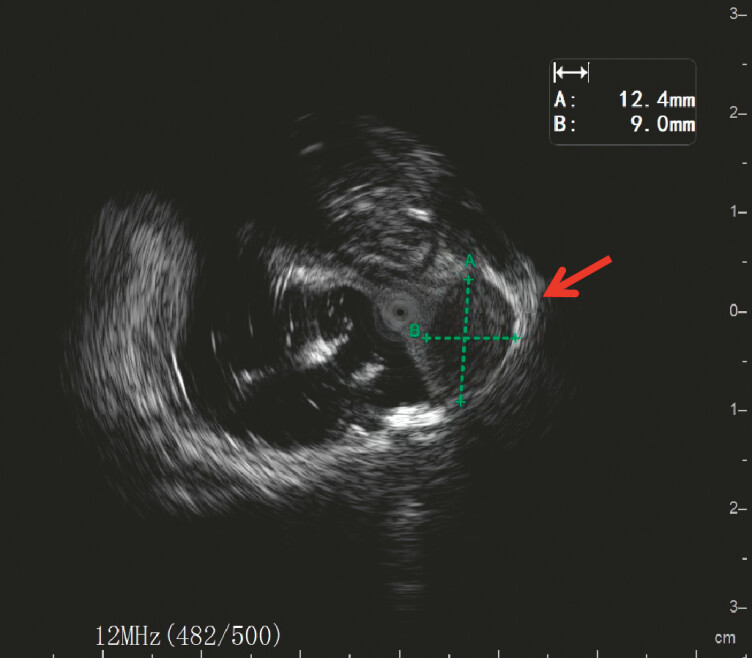
An endoscopic ultrasound image showing a hypoechoic mass within the muscularis propria of the bulb. The lesion demonstrates homogeneous echogenicity, intramural and extramural growth, and well-defined borders and measures approximately 12 × 9 mm (arrow).

A 56-year-old woman was found to have a submucosal tumor in the duodenal bulb during a routine gastroscopy. She was asymptomatic. Her medical history was unremarkable. Contrast-enhanced computed tomography revealed a well-defined, markedly enhancing nodule (approximately 12 mm in diameter) at the superior aspect of the duodenal bulb ( Fig. 1 ). Endoscopic ultrasound revealed a 12mm × 9 mm hypoechoic mass within the muscularis propria of the bulb, with homogeneous echogenicity, intramural and extramural growth, and well-defined borders ( Fig. 2 ).

**Fig. 3:**
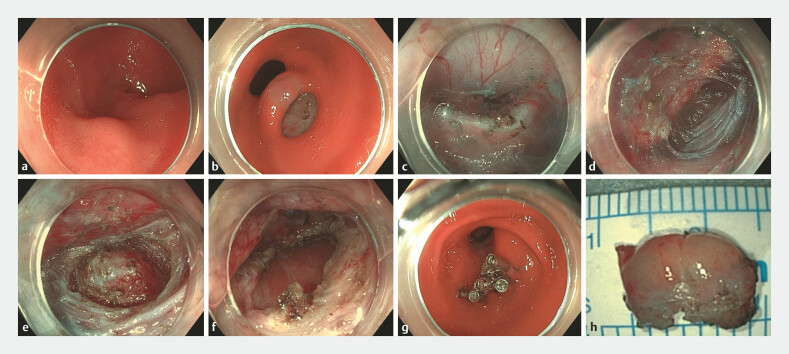
Endoscopic submucosal tunneling resection via a transantral approach for a duodenal bulb submucosal tumor. **a** An endoscopic view of the submucosal tumor at the duodenal bulb. **b** Creation of the submucosal tunnel from the gastric antral orifice toward the duodenal bulb. **c** Prior to myotomy of the pyloric sphincter. **d** Limited myotomy of the pyloric sphincter during tunnel advancement. **e** Dissection of the tumor from the muscularis propria within the established tunnel. **f** Resection bed within the duodenal bulb tunnel following tumor removal. **g** Closure of the gastric tunnel orifice with metallic clips. **h** The resected specimen measuring approximately 15 × 10 mm.

Given the high-risk anatomy of the duodenal bulb, including its rich vasculature, thin muscular layer, and narrow lumen, an endoscopic transantral submucosal tunneling approach was employed to resect the lesion ( Video 1 ). Key procedural steps included tunnel creation from the gastric antrum, limited myotomy across the pylorus, and en bloc tumor resection within the tunnel ( Fig. 3 ). The resection was complete, with no significant intraoperative bleeding or perforation.

Endoscopic submucosal tunneling resection via a transantral approach for the duodenal bulb gastrointestinal stromal tumor.Video 1

**Fig. 4:**
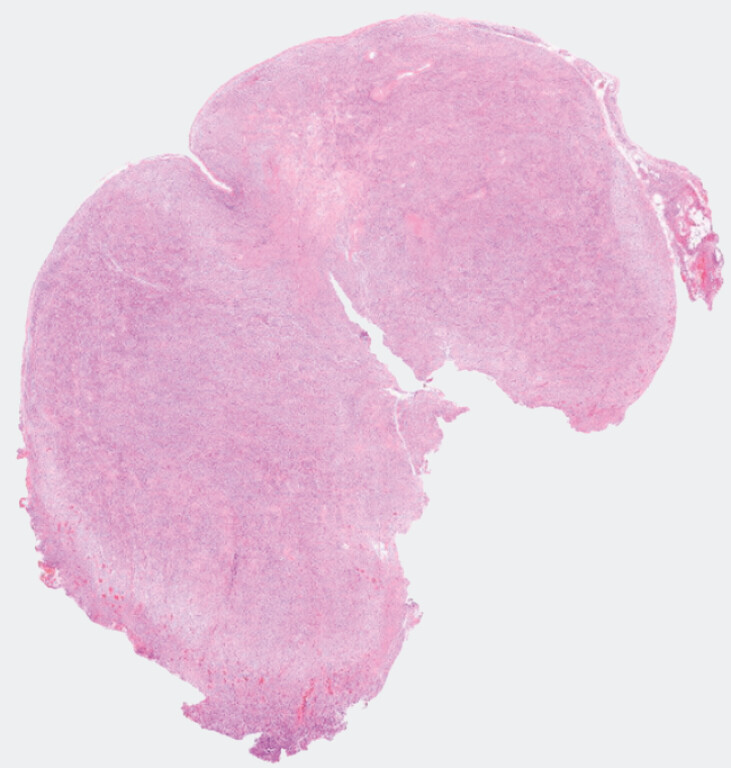
Histopathological examination (H & E stain) of the resected specimen confirms the diagnosis of the gastrointestinal stromal tumor (GIST).

Histopathological examination of the resected specimen confirmed the diagnosis of a gastrointestinal stromal tumor, classified as very low risk ( Fig. 4 ). Immunohistochemistry showed negative S-100 and Desmin, but positive CD117 and DOG-1, and a Ki-67 proliferation index of approximately 2%.

The patient had an uneventful recovery. She was discharged on postoperative day 6 and remained asymptomatic at 1-month follow-up. This case demonstrates that transantral submucosal tunneling resection is a feasible, safe, and organ-preserving endoscopic technique for selected submucosal tumors in the challenging location of the duodenal bulb, offering a minimally invasive alternative to surgical intervention.

Endoscopy_UCTN_Code_TTT_1AO_2AG

